# Wild-type but not mutant huntingtin modulates the transcriptional activity of liver X receptors

**DOI:** 10.1136/jmg.2009.066399

**Published:** 2009-05-17

**Authors:** M Futter, H Diekmann, E Schoenmakers, O Sadiq, K Chatterjee, D C Rubinsztein

**Affiliations:** 1 CIMR, Medical Genetics, Wellcome Trust/MRC Building, Addenbrooke’s Hospital, Cambridge, UK; 2 Summit plc, Abingdon, Oxfordshire, UK; 3 Department of Medicine, University of Cambridge, Cambridge, UK

## Abstract

**Background:**

Huntington’s disease is caused by expansion of a polyglutamine tract found in the amino-terminal of the ubiquitously expressed protein huntingtin. Well studied in its mutant form, huntingtin has a wide variety of normal functions, loss of which may also contribute to disease progression. Widespread transcriptional dysfunction occurs in brains of Huntington’s disease patients and in transgenic mouse and cell models of Huntington’s disease.

**Methods:**

To identify new transcriptional pathways altered by the normal and/or abnormal function of huntingtin, we probed several nuclear receptors, normally expressed in the brain, for binding to huntingtin in its mutant and wild-type forms.

**Results:**

Wild-type huntingtin could bind to a number of nuclear receptors; LXRα, PPARγ, VDR and TRα1. Over-expression of huntingtin activated, while knockout of huntingtin decreased, LXR mediated transcription of a reporter gene. Loss of huntingtin also decreased expression of the LXR target gene, ABCA1. In vivo, huntingtin deficient zebrafish had a severe phenotype and reduced expression of LXR regulated genes. An LXR agonist was able to partially rescue the phenotype and the expression of LXR target genes in huntingtin deficient zebrafish during early development.

**Conclusion:**

Our data suggest a novel function for wild-type huntingtin as a co-factor of LXR. However, this activity is lost by mutant huntingtin that only interacts weakly with LXR.

Huntington’s disease (HD) is a devastating autosomal dominant neurodegenerative disease characterised by motor, psychiatric and cognitive dysfunctions. HD is caused by an abnormal expansion of a polyglutamine tract located in the N-terminus of a ubiquitously expressed protein called huntingtin.^1^ Huntingtin polyglutamine tracts in the normal population have up to 35 CAG repeats while those of HD patients are expanded to 36 or more CAG repeats.^2^ Genetic data in humans and transgenic animal models suggest that polyglutamine expansions confer a deleterious gain-of-function on the target proteins.^3^ However, this does not preclude the possibility that the severity of HD may be modified by loss-of-function effects.^4^ Huntingtin is present in the nucleus, suggesting it may play a role in nuclear events.^5^ Single candidate gene and unbiased microarray approaches have clearly demonstrated that transcription of numerous genes is dysregulated in human HD brain^6^ and in transgenic mouse and cell models of HD.^7^ The promoter motifs of genes downregulated in HD cell models suggested defects in transcription mediated through cAMP response element binding (CREB) protein, retinoic acid receptor (RAR), and specificity protein-1 (Sp1).^8^ ^9^

Both wild-type and mutant huntingtin bind to a number of transcription factors, including Sp1 and CREB protein. In some cases, this interaction can be strengthened by expanded polyglutamine.^10^^–^12 Mechanistically, huntingtin appears to regulate transcription both as a normal function and as a deleterious gain-of-function upon expansion of its polyglutamine tract. Although transcription factors can be localised to nuclear inclusions formed by mutant huntingtin,^12^^–^14 it is unclear whether mutant huntingtin perturbs transcription by simply sequestering transcription factors in aggregates.^15^ Soluble mutant huntingtin may act to disrupt transcription factor binding to DNA.^10^ ^11^ Important work has revealed a role for wild-type huntingtin in HD where loss of function of huntingtin contributes to the severity of the disease by disrupting transcription of neuronal genes. Wild-type but not mutant huntingtin stimulated transcription of the gene encoding brain derived neurotrophic factor (BDNF).^4^ This effect occurs through cytoplasmic sequestering of repressor element-1 transcription factor/neuron restrictive factor (REST/NRSF) that can bind to the promoter of the BDNF gene to repress its transcription. Mutant huntingtin binds less avidly to REST/NRSF allowing it to enter the nucleus and repress BDNF gene expression.^16^ Polyglutamine expansion may result in both a loss and a gain of specific functions of a protein, as has been elegantly demonstrated for ATX1, where expansion of the CAG repeat differentially affects its function in the context of different endogenous protein complexes.^17^

To elucidate new transcriptional pathways regulated by huntingtin, we tested several nuclear receptors (NRs), normally expressed in the brain, for their ability to interact with the N-terminal fragment of huntingtin (amino acids 1–588) with a normal (17Q) or expanded (138Q) polyglutamine repeat. Nuclear receptors are of particular interest since they control a wide range of physiological processes and are highly expressed in the brain; 94% of all nuclear receptors are found endogenously in neurons.^18^ In this study, we found wild-type huntingtin could bind to a number of nuclear receptors including: LXRα, peroxisome proliferator-activated receptor-γ (PPARγ), vitamin D receptor (VDR), and thyroid hormone receptor-α1 (TRα1). Recent work describes the disruption of cholesterol homeostasis in HD.^19^ The two LXRs, LXRα and LXRβ, are recognised to be central regulators of cholesterol metabolism in mammals. Both LXRs activate target genes by binding to response elements located in their promoter regions as heterodimers with the retinoid X receptor (RXR). Oxysterols and intermediates of the biosynthetic cholesterol pathway have been identified as the natural ligands for LXR (reviewed by Wojcicka *et al*^20^). We found LXR bound to wild-type huntingtin via its ligand binding domain (LBD). Over-expression of huntingtin activates LXR mediated transcription and knockout of huntingtin decreases LXR mediated transcription. Furthermore, knockout of huntingtin decreases expression of ABCA1, a key downstream target gene regulated by LXR. An agonist of LXR partially rescues the phenotype observed in huntingtin deficient zebrafish. These data suggest that huntingtin can regulate LXR mediated transcription and may play a general role as a co-factor of nuclear receptors. LXR transcriptional deficiency may contribute to the phenotypes of huntingtin defects in vivo.

## METHODS

### Expression constructs

cDNAs encoding human LXRα, PPARγ, VDR, TRα1 and RXRα were polymerase chain reaction (PCR) cloned into the pcDNA3.1Myc-His vector using the following restriction sites respectively; BamHI/XhoI, KpnI/XhoI, BamHI/XhoI, BamHI/NotI and EcoRI/XhoI. GST tagged LXRα was then generated by subcloning into BamHI/XhoI digested pGEX-6P-1. His tagged huntingtin 190 was generated by PCR cloning into NdeI/BamHI digested pET15b. Leucine to alanine point mutations were generated using the Quikchange (Stratagene, La Jolle, California, USA) site directed mutagenesis kit. All constructs were verified by DNA sequencing.

### Immunoprecipitations

SK-N-SH or COS-7 cells were plated in 10 cm dishes and transfected with 2 μg Myc tagged NR constructs and 2 μg FLAG tagged Htt constructs as specified in the text, using either Lipofectamine Plus (Invitrogen, Paisley, UK) or Lipofectamine (Invitrogen) (20 μl Plus and 10 μl lipofectamine per dish), respectively. The medium was changed after 4 h. Following 24 or 48 h incubation, respectively, cells were lysed in IP buffer (20 mM Tris pH7.5, 150 mM NaCl, 0.5% NP40, 2 mM MgCl_2_, protease inhibitors) and protein assays performed (Dc protein assay, Bio-rad, Hemel Hempstead, UK). 100 μg total protein was incubated with rabbit anti-Myc antibody overnight on a rotating shaker at 4°C. A volume of 15 μl protein A magnetic beads (Invitrogen) per IP was washed once in IP buffer, resuspended to 15 μl then added to the lysate/antibody mix and incubated for a further 1 h. The beads and proteins bound were then separated from the remaining lysate using a magnet and were washed three times in IP buffer. The bound proteins were eluted in 100 mM glycine pH2.5, resolved by SDS-PAGE and transferred to PVDF membrane (Anachem, Luton, UK). Membranes were probed with anti-huntingtin monoclonal antibody (Chemicon, Millipare, Watford, UK) or anti-FLAG M2 antibody (Sigma, St Louis, Missouri, USA) to detect huntingtin immunoprecipitated by NRs.

### Luciferase assays

HEK cells or ES cells were plated on poly-D-lysine or gelatin coated 96 well plates. HEK cells were transfected with 1.7 ng LXR, 1.7 ng LXR response element (LXRE) or empty luciferase reporter, 5 ng β-galactosidase, 51 ng Htt or empty vector, 0.6 μl Plus reagent and 0.3 μl lipofectamine reagent per well. ES cells were transfected with 40 ng LXR, 40 ng LXRE, 120 ng β-galactosidase and 0.5 μl lipofectamine 2000 per well. The medium was changed after 4 h incubation. Following 24 h incubation, luciferase assays were performed. Cells were treated overnight with 50 nM of the LXR agonist, TO901317, as indicated.

### Q-PCR

ES cells were incubated overnight (18 h) in the presence or absence of 25 nM LXR agonist, GW683965A. Zebrafish were injected with huntingtin morpholino and treated with LXR agonist as described below. RNA was prepared from ES cells or 72 hpf zebrafish using the RNAeasy kit (Qiagen, Crawley, UK). Following DNase treatment (Invitrogen), 1 μg RNA was reverse transcribed to make cDNA using the Superscript III kit (Invitrogen). Q-PCR was performed using 8 μl cDNA, 375 nM final concentration of each primer and 2X SYBR green mastermix (ABI systems, Warrington, UK) in a total of 20 μl per well on an ABI 7900 fast Q-PCR machine. Cts were normalised to a cDNA standard curve and to the housekeeping gene GAPDH or β-actin. Primers used were: ABCG1 5′- CAGGGTGGAAGAACCGTCAT -3′ and 5′- AAGCTTGTCGAAGAGCTCGAA-3′, CYP7A1 5′- GCAGGCGTGCCAATGTG -3′ and 5′- CCAACTGCTCCCTTGTCAGT-3′, LXR 5′- GAGATTCTCAGTCAAACGGACTTG-3′ and 5′- TGATGTCGTTGGATTCCATGA-3′, FASN 5′- CGCTTGTCCTACTTCTTTGATTTCA-3′ and 5′- TTCCAGTGCCAGCAGACTAGAG-3′, ACC 5′- GTGGAGAAGGCCATCAAAAA-3′ and 5′- TGCTGAAACTGAACCTCCACT-3′, β-actin 5′- TGCCCCTCGTGCTGTTTT-3′ and 5′- TCTGTCCCATGCCAACCAT-3′, GAPDH 5′- TGTGTCCGTCGTGGATCTGA-3′ and 5′- CCTGCTTCACCACCTTCTTGAT-3′, ABCA1 5′- AAGGGTTTCTTTGCTCAGATTGTC -3′ and 5′- TGCCAAAGGGTGGCACA-3′

### GST and His tagged protein purification and GST pulldowns

GST and His tagged proteins were purified from BL21 *Escherichia coli*; 200 ml cultures (OD 0.8) were induced with 1 mM IPTG for 1 h, pelleted and resuspended in 20 ml GST (10 mM KCl, 1 mM DTT, protease inhibitors in PBS) or His binding buffer (50 mM NaH_2_PO_4_, 300 mM NaCl, 10 mM imidazole). Lysozyme was added to a final concentration of 1 mg/ml and the cell suspension incubated for 15 mins at room temperature. The suspension was then lysed by five freeze-thaw cycles and NP40 added to a final concentration of 0.5%. Following 30 mins incubation the lysate was sonicated (three bursts for 30 s each) using a probe sonicator and insoluble material removed by centrifugation at 10 000 rpm for 20 mins. GST or His tagged proteins were batch purified with 0.5 ml GST-sepharose (Amersham Biosciences) or 0.5 ml Ni-NTA agarose (Qiagen), washed four times in GST binding buffer or His washing buffer (50 mM NaH_2_PO_4_, 300 mM NaCl, 20 mM imidazole) and eluted with 10 mM glutathione or 250 mM imidazole. Purified proteins were extensively dialysed into PBS.

GST pulldowns were set up using 0.5 μg of either GST or GST tagged LXR and 20 μl glutathione sepharose beads diluted in 1 ml GST binding buffer and incubated for 30 mins. 0.5 μg His tagged Htt 190 was added and the tubes incubated for a further 2 h. Protein bound to the beads were separated using spin columns to collect the beads followed by three washes in GST binding buffer and one wash in PBS then elution in 2X SDS sample buffer.

### Zebrafish strain husbandry

Wild type zebrafish of the AB strain were maintained at 28.5°C under standard conditions in compliance with UK Home Office regulations. Embryos were collected after natural spawning, staged as previously described^21^ and raised in embryo medium.

### Morpholino injections and LXR treatment

A morphilino, Htt_ATG, was designed to specifically target the zebrafish *huntingtin* translation initiation codon (ATG targeting: 5′-GCC ATT TTA ACA GAA GCT GTG ATG A- 3′ (+5 to −20); obtained from Gene Tools, Philomath, Oregon, USA) and dissolved in 1× Danieau medium (58 mM NaCl, 0.7 mM KCl, 0.4 mM MgSO4, 0.6 mM Ca(NO_3_), 2, 5 mM HEPES, pH 7.6). Morpholinos with the same sequence have already been used and proved to be specific for the downregulation of huntingtin expression in zebrafish.^22^ As a negative control, a mispair control morpholino with five base modifications out of 25 (Htt_ATG-5mis: 5′-GCg Ata TTA ACA cAA cCT GTc ATG A- 3′) was used and did not produce any phenotype upon injection. Zebrafish eggs (1–2 cell stage) were injected with 3.5 ng Htt_ATG morpholino or 3.5 ng corresponding 5 bp mismatch control (Htt_ATG-5mis) morpholino. At 7 hpf (for cartilage staining) or 86 hpf (for mRNA expression), half of the injected embryos were placed in E3 medium (5 mM NaCl, 0.17mM KCl, 0.33 mM CaCl2.2H2O, 0.33 mM MgSO4.7H2O, 5 mM HEPES) supplemented with 200 nM TO901317 LXR agonist (Tocris, Bristol, UK) and zebrafish were raised at 28.5°C until 104 hpf.

### Zebrafish cartilage staining

After fixation in 4% PFA, 104 hpf zebrafish were stained in Alcian green (0.1% w/v in 0.37% HCl/70% EtOH) at room temperature overnight. Zebrafish were washed in 0.37% HCl/70% EtOH, rehydrated and digested with trypsin for ∼4 h at 37°C. Pigment was bleached in 3% H_2_O_2_ in 0.5% KOH for ∼30 min and fish were stored in 100% glycerol at 4°C. Lower jaws were dissected using Tungsten needles and photographed with an Olympus SZX12 microscope at the same magnification.

## RESULTS

### Huntingtin binds to nuclear receptors

**Figure 1:**
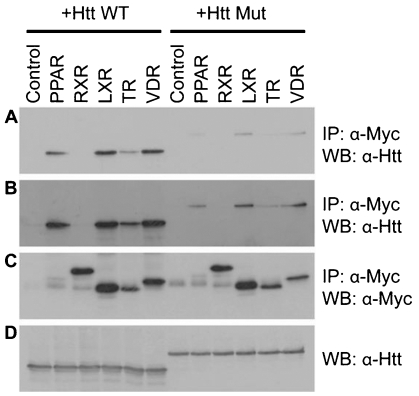
Huntingtin binds to nuclear receptors. Immunoprecipitation of huntingtin from COS-7 cells transiently expressing pcDNA3.1 Myc-His empty vector (control) or each of the following Myc tagged receptors; PPARγ, RXRα, LXRα, TRα1 and VDR in combination with either wild-type or mutant huntingtin 1–588. Representative blots show Myc antibody immunoprecipitations probed with huntingtin antibody (a short exposure in panel A and a longer exposure in panel B), Myc antibody immunoprecipitations probed with Myc antibody (C) and FLAG tagged huntingtin expressed in total lysate (D).

With the aim of identifying new transcriptional pathways regulated by huntingtin, we tested the following brain expressed nuclear receptors; LXRα, PPARγ, VDR and TRα1 and RXRα for binding to wild-type and mutant huntingtin. Myc-tagged LXRα, PPARγ, VDR, TRα1 and RXRα and FLAG tagged huntingtin 588 (amino acids 1–588) were expressed in SK-N-SH cells. The NRs were immunoprecipitated using anti-Myc antibody and any associated huntingtin was detected by western blot probed with anti-huntingtin antibody. LXRα, PPARγ, VDR and TRα1 but not RXRα were able to bind to wild-type huntingtin (fig 1A). Much lower amounts of LXRα, PPARγ, VDR and TRα1 were bound to mutant huntingtin (in long exposures of blots, fig 1B). Control blots show that similar amounts of wild-type and mutant huntingtin were expressed (fig 1D) and that similar amounts of LXRα, VDR, TRα1 and RXRα were immunoprecipitated (fig 1C). PPARγ was expressed at a lower level in total lysate (data not shown) and therefore less PPARγ is immunoprecipitated relative to the other nuclear receptors. These data suggest wild-type huntingtin binds to nuclear receptors to a region conserved between LXRα, PPARγ, VDR and TRα1 but not RXRα and that mutation of huntingtin’s polyglutamine tract interferes with this interaction.

### Loss of endogenous huntingtin impairs LXR activated transcription

**Figure 2:**
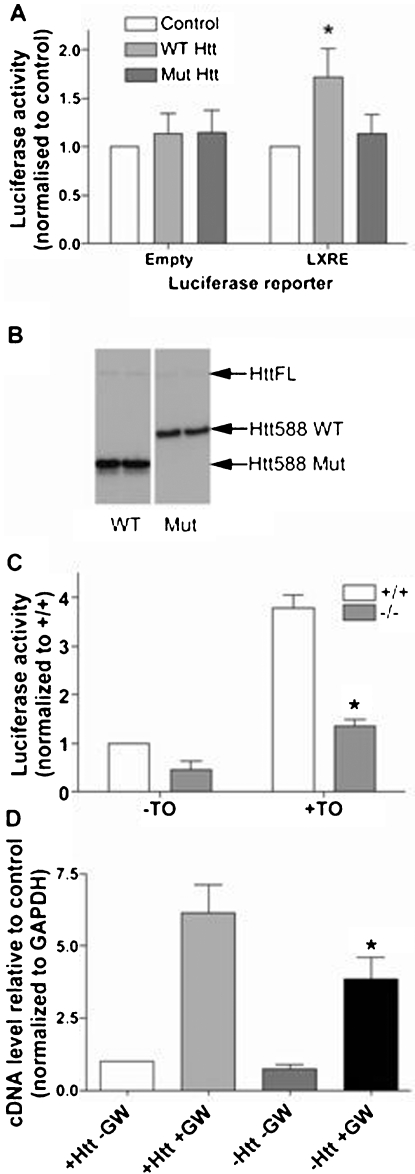
Huntingtin activates LXR dependent gene transcription. (A) Activity of an LXRE-luciferase or empty luciferase reporter expressed in HEK cells in combination with LXR and Htt (wild-type and mutant). Luciferase activity was normalised to β-galactosidase activity and compared to empty (no Htt) vector control (mean ±SEM, n = 3 experiments; *p<0.05 as analysed by one way analysis of variance (ANOVA) with Dunnett’s post test compared to empty vector control). (B) Western blot showing relative expression of endogenous huntingtin and transiently expressed huntingtin 1–588 wild-type (WT) and mutant (Mut) in HEK cells. (C) Activity of an LXRE-luciferase reporter expressed in *Hdh^ex4/5^/Hdh^ex4/5^* knockout (−/−), and wild-type (+/+) embryonic stem cells in the presence of the LXR agonist TO901317 (TO). Luciferase activity was normalised to β-galactosidase activity and plotted compared to wildtype -TO (mean ±SEM, n = 6 experiments; luciferase activity in the presence of agonist in −/− cells is significantly different from +/+ cells; *p<0.05 as analysed by one way ANOVA with Dunnett’s post test). (D) QPCR assessment of expression of ABC-A1 mRNA in *Hdh^ex4/5^/Hdh^ex4/5^* knockout (−/−) and wild-type (+/+) ES cells following induction of LXR activity by the LXR agonist GW683965A (GW). cDNA levels were normalised to GAPDH values and plotted compared to +/+ -GW (mean ±SEM, n = 4 experiments; ABC-A1 levels in agonist treated knockout cells are significantly different from agonist treated wild-type cells; *p<0.05 as analysed by one way ANOVA with Dunnett’s post test).

Recent work describes the disruption of cholesterol homeostasis in HD.^19^ Cholesterol homeostasis is controlled by LXR regulation of transcription, therefore we investigated whether huntingtin was able to regulate LXR activated transcription. Wild-type or mutant huntingtin aa1-588 were expressed in combination with an LXR response element (LXRE) upstream of a luciferase gene (LXRE-luciferase reporter), or empty luciferase reporter, LXR and β-galactosidase. Transcriptional activity from the LXRE-luciferase or empty reporter was measured following induction by the LXR agonist TO901317. Wild-type huntingtin significantly increased LXR activated transcription by 72% but had no effect on an empty luciferase reporter (fig 2A). Mutant huntingtin had no significant effect on LXR activated transcription, consistent with its weak LXR binding ability (fig 2A). In these experiments, huntingtin 588 was expressed at much higher levels than endogenous full length huntingtin as shown by western blot (fig 2B).

To test whether loss of huntingtin decreased LXR activated transcription, we studied embryonic stem cells from wild-type mice or huntingtin knockout mice.^23^ An LXRE-luciferase reporter, LXR and β-galactosidase were transiently expressed in these cells, transcriptional activity measured using a luciferase assay and normalised for differences in expression levels by β-galactosidase assay. In the presence of LXR agonist, LXR activated transcription was decreased by 64% in huntingtin knockout cells compared to huntingtin wild-type cells (fig 2C). Thus, cells expressing normal levels of wild-type full length huntingtin have higher LXR mediated transcriptional activity, compared to knockout cells, confirming the physiological importance of huntingtin in this process.

LXR has been shown to activate transcription of a number of genes involved in cholesterol metabolism including the ATP binding cassette transporter 1 gene (ABCA1). The ABCA1 protein mediates the first step of cholesterol reverse transport, eliminating excess cholesterol from the cell.^24^ To determine whether huntingtin could modulate LXR activated transcription of endogenous genes, we performed Q-PCR to assess the mRNA levels of ABCA1 transcribed following LXR activation with LXR agonist GW683965 treatment in huntingtin knockout cells. As expected, ABCA1 mRNA levels were significantly increased by LXR activation in wild-type cells. This increase was significantly attenuated in agonist-treated huntingtin knockout cells to (mean (SEM)) 3.9 (0.7) from 6.2 (1.0) (fold increase over untreated cells) in agonist treated huntingtin wild-type cells (Fig. 2D). Similar results were seen with another LXR agonist, TO901317 (data not shown). LXRα and LXRβ gene transcription was not significantly different in the two cell lines (data not shown). These data suggest wild-type huntingtin is able to act as a co-factor of LXR mediated transcription since overexpression of huntingtin 588 increases LXR mediated transcription of a reporter gene and knockout of huntingtin decreases LXR mediated transcription of a reporter and a target gene. In addition, expansion of the polyglutamine tract in mutant huntingtin leads to loss of Huntington’s co-factor function.

### Mapping the huntingtin-LXR regions of interaction

**Figure 3:**
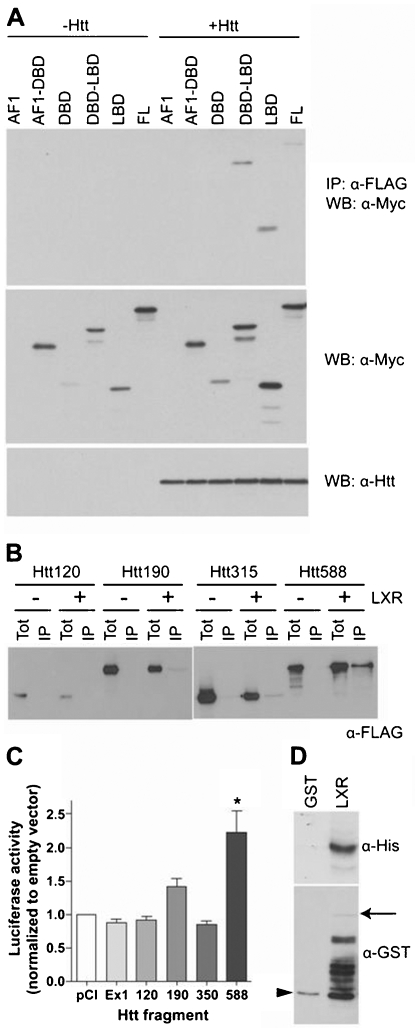
N-terminal huntingtin region 1–588 and ligand binding domain (LBD) of LXR are necessary for the huntingtin-LXR interaction. (A) Domains of LXR that bind to huntingtin. Myc-tagged regions of LXR; AF1, AF1-DNA-binding domain (AF1-DBD), DBD, DBD-ligand binding domain (DBD-LBD), LBD and full length (FL) were expressed in COS-7 cells in combination with FLAG-tagged huntingtin 1–588 (middle and bottom panels, respectively). AF1 was expressed at much lower levels than the other fragments. Immunoprecipitations were performed with FLAG antibody and binding proteins were detected by Myc antibody (top panel). (B) Region of huntingtin that binds to LXR. FLAG tagged regions of huntingtin: 1–120 (Htt120), 1–190 (Htt190), 1–315 (Htt315), 1–588 (Htt588) were expressed in SK-N-SH cells in combination with Myc-tagged LXR. Immunoprecipitations were performed with Myc antibody and binding proteins were detected by FLAG antibody. (C) Activity of an LXRE-luciferase reporter in HEK cell lysates expressing different constructs of huntingtin; 1–120 (Htt120), 1–190 (Htt190), 1–315 (Htt315) and 1–588 (Htt588). Luciferase activity was normalised to β-galactosidase activity and plotted compared to empty vector control (emp) (mean ±SEM, n = 3 experiments; *p<0.05 as analysed by one way ANOVA with Dunnett’s post test compared to empty vector control). (D) GST pulldown of purified His tagged huntingtin 1–190 by GST tagged LXR. Arrow points to GST-LXR and arrowhead points to GST.

NRs have a conserved domain structure consisting of an N-terminal activation-function domain (AF-1), a DNA binding domain (DBD) containing two zinc fingers, a flexible hinge domain and the ligand binding domain (LBD) whose C-terminal end also has transcriptional activation function(s).^20^ To map the region(s) of LXR that bind to huntingtin, we made Myc tagged constructs encompassing the various LXR domains; AF-1 (aa1-91), AF-1/DBD (aa1-263), DBD (aa92-263), DBD/LBD (aa92-447) and LBD (aa263-447) and transiently expressed them with FLAG-tagged huntingtin 588 in SK-N-SH cells. The LXR domains were immunoprecipitated using anti-Myc antibody and any associated Htt was detected by western blot probed with anti-huntingtin antibody. The DBD-LBD and LBD fragments but not the other fragments of LXR, bound strongly to huntingtin demonstrating that the LBD domain is sufficient for the interaction with huntingtin to occur (fig 3A).

To dissect the region of huntingtin that was important for its interaction with LXR, we made FLAG tagged deletion constructs of huntingtin;^25^ Htt120 (aa1-120), Htt190 (aa1-190) and Htt315 (aa1-315). We expressed each of these huntingtin fragments, including Htt588, in combination with Myc-tagged LXR in COS-7 cells. LXR was immunoprecipitated using anti-Myc antibody and any associated huntingtin was detected by western blot probed with anti-FLAG antibody. Htt588 bound strongly to LXR while Htt190 and Htt315 bound more weakly. We were unable to detect Htt120 bound to LXR, but this may be because Htt120 was expressed at lower levels than Htt190, Htt315 and Htt588 (fig 3B). Therefore, it appears that the entire 1–588 region of huntingtin is necessary for strong binding to LXR.

We also tested whether these huntingtin fragments could activate LXR mediated transcription in a similar manner to Htt588. An LXRE-luciferase reporter, LXR and β-galactosidase were transiently expressed in HEK cells in combination with either GFP-HttExon1, FLAG-Htt120, FLAG-Htt190, FLAG-Htt350 or FLAG-Htt588. Transcriptional activity was measured using a luciferase assay and normalised for differences in expression levels by β-galactosidase assay. As previously shown in fig 3A, Htt588 was able to activate LXR mediated transcription. However, the other huntingtin fragments, HttExon1, Htt120, Htt190 and Htt 315, were unable to significantly alter LXR mediated transcription (fig 3C). Thus, huntingtin fragments and mutant huntingtin that bind LXR weakly do not activate LXR mediated transcription.

Our experiments demonstrate that LXR and huntingtin interact. This interaction may be direct or may be mediated by another protein or complex of proteins. Therefore, we tested whether huntingtin could interact directly with LXR. GST tagged LXR and His tagged Htt190 were purified from *E coli* and their in vitro binding tested using a GST pulldown assay. His-Htt190 was pulled down by GST-LXR but not GST alone, demonstrating that Htt and LXR can interact directly in vitro (fig 3D).

We attempted to test whether endogenous LXR in the brain could interact with endogenous huntingtin. LXR is important for brain function, and knockout of both LXRα and LXRβ resulted in severe abnormalities of the brain and neurodegeneration.^26^ Most areas of the brain have low-medium levels of LXRα mRNA and medium-high levels of LXRβ mRNA^18^; however, LXR protein was barely detectable in the brain (see fig S1A, B in supplemental material). We also tried to test for an interaction between the endogenous proteins in several different cell lines but again LXR was barely detectable (supplemental fig S1B). LXR is highly expressed in the liver, but unfortunately huntingtin is poorly expressed in the liver (supplemental fig S1A). Thus, detection of an endogenous interaction between LXR and huntingtin has not proved to be possible, as even a 10% interaction would be far below the limits of detection in our system. It is important to note that we are unaware of successful endogenous LXR immunoprecipitation to detect interacting proteins in the literature, presumably due to its low abundance. Furthermore, the entire literature examining the effect of LXR interacting proteins on its transcriptional activity have relied on LXR over-expression experiments to dissect the interactions and transcriptional effects.^27^ ^28^

### The huntingtin-LXR interaction is not mediated by pentapeptide LxxLL motifs present in huntingtin

**Figure 4:**
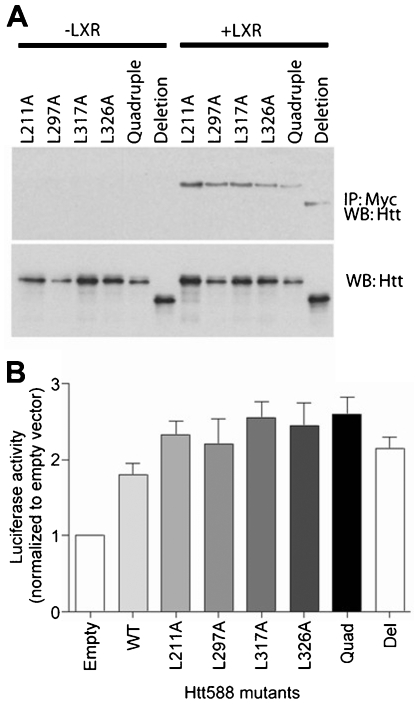
Mutation of nuclear receptor binding motifs in huntingtin has no effect on transcriptional activation. (A) Immunoprecipitation of Myc-tagged LXR and (B) activity of an LXRE-luciferase reporter expressed in SK-N-SH cells or HEK cells, respectively, in combination with individual Leu to Ala FLAG-huntingtin 1–588 mutants (L211A, L297A, L317A, L326A), a mutant with all five NR binding motifs mutated (quadruple) or a deletion mutant missing the region containing the five motifs (deletion).

The LBD of NRs contains a shallow hydrophobic groove that crystallographic studies have shown to bind a pentapeptide motif (LxxLL) found in a number of co-activator proteins (NR binding motif).^29^ Huntingtin 588 contains five of these motifs, two of which share a common leucine. To determine whether any of these NR binding motifs could mediate the interaction between huntingtin and the LXR LBD, we made a series of leucine to alanine point mutations (L211A, L297A, L317A and L326A). We also made a mutant containing all four point mutations (quadruple) and a deletion mutation (deletion) encompassing the entire LxxLL motif-containing region. To test whether these mutants were still able to bind LXR we expressed each mutant with or without LXR, immunoprecipitated LXR using a Myc antibody and detected any associated huntingtin by western blot probed with anti-huntingtin antibody. All the mutants were able to bind LXR suggesting that none of the NR binding motifs were necessary for the huntingtin-LXR interaction (fig 4A). We also expressed each of these mutants in combination with an LXRE-luciferase reporter, LXR and β-galactosidase in HEK cells. Transcriptional activity was measured using a luciferase assay and normalised for differences in expression levels by β-galactosidase assay. Consistent with the interaction data, all the mutants were able to activate LXR mediated transcription in a manner similar to Htt588 (fig 4B). There were no significant differences between the mutants and wild-type Htt588 as assessed by analysis of variance (ANOVA). These data suggest that the LxxLL motifs are not necessary for the huntingtin-LXR interaction or for activation of LXR mediated transcription.

### An LXR agonist partially rescues zebrafish huntingtin knockdown phenotype

**Figure 5:**
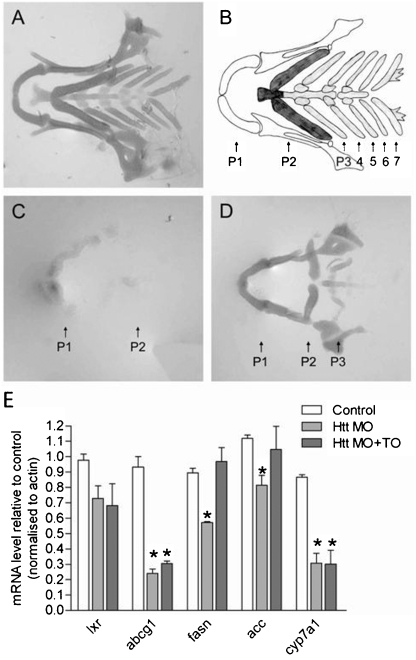
Effect of an LXR agonist on cartilage of Htt-deficient zebrafish. (A) Ventral view of an Alcian green stained lower jaw of an Htt_ATG-5mis control at 102 hpf depicting the normal zebrafish cartilage. A representative zebrafish is shown from five independent experiments where 11–22 zebrafish were analysed per experiment. (B) Diagram of the pharyngeal skeleton in ventral view showing the first or mandibular arch (P1, white), the second or hyoid arch (P2, dark grey) and five brachial arches (P3–7, light grey). (C) Cartilage is strongly reduced in Htt_ATG injected zebrafish. The mandibular (P1) and hyoid (P2) are present but small and malformed and pharyngeal arches P3–5 are missing. A representative zebrafish is shown from five independent experiments where 7–20 zebrafish were analysed per experiment. (D) An LXR agonist rescues formation (compactation, form, staining intensity) of the anterior two arches. A representative zebrafish is shown from fove independent experiments where 9–14 zebrafish were analysed per experiment. (E) Q-PCR assessment of expression of LXR and LXR target genes mRNA in uninjected control zebrafish and Htt MO injected zebrafish at 3 dpf. cDNA levels were normalized to actin levels and were plotted compared to control zebrafish (mean ±SEM, n = 3−4 experiments; *p<0.05 as analysed by one way ANOVA with Dunnett’s post test).

To investigate whether regulation of LXR mediated transcription by wild-type huntingtin could play a role in vivo, we tested whether activation of LXR could rescue development in huntingtin deficient zebrafish. Blocking translation of huntingtin mRNA using ATG specific morpholino oligonucleotides resulted in severe reduction of cartilage in the lower jaw.^30^ Wild-type zebrafish have seven pharyngeal arches present as represented diagrammatically in fig 5A,B. In control morpholino injected, as in wild-type zebrafish, seven pharyngeal arches are observed (fig 5A). Huntingtin mRNA is expressed in the cartilage.^30^ When huntingtin was knocked down in huntingtin-ATG injected zebrafish larvae, only the mandibular and hyoid arches (P1 and P2) are detectable and they are reduced in size and wrongly orientated (fig 5C). Either all or the last four of the five branchial arches are missing and overall staining intensity is reduced compared to controls, indicating poor cartilage development. Treatment of morpholino injected zebrafish with LXR agonist TO901317 did not significantly restore the missing branchial arches (fig 5D). However, intensity of the staining and size of the remaining mandibular and hyoid arches is visibly increased, indicating rescue of cartilage formation in huntingtin deficient zebrafish. The LXR agonist had no effect on control zebrafish.

Only one isoform of LXR is expressed in zebrafish. This isoform has a higher similarity with mammalian LXRα, although its ubiquitous expression pattern more closely resembles LXRβ.^31^ In zebrafish embryos, LXR is expressed in the first 24 h following fertilisation suggesting a role for LXR during development.^31^ In adult zebrafish, as in mammalians, LXR is likely to be involved in regulation of lipid and cholesterol homeostasis. LXR agonists have been demonstrated to activate a number of zebrafish LXR target genes involved in these processes including ATP binding cassette transporter G1 (ABCG1), cytochrome P450 a1 (CYP7A1), fatty acid synthase (FASN) and acetyl-CoA carboxylase (ACC).^31^ We had observed a partial rescue of a zebrafish huntingtin knockdown phenotype upon treatment with an LXR agonist and therefore investigated whether expression of these zebrafish LXR target genes could be regulated by huntingtin knockdown and subsequent rescue with LXR agonist. Expression of LXR and LXR target gene mRNAs were assessed in uninjected control zebrafish and huntingtin morpholino injected zebrafish treated with and without LXR agonist. mRNA levels of all four LXR target genes tested, ABCG1, CYP7A1, FASN and ACC, were significantly downregulated following knockdown of huntingtin, consistent with our previous observations that wild-type huntingtin activates transcription of LXR target genes (fig 5E). Treatment of huntingtin morpholino injected zebrafish with LXR agonist TO901317 rescued the downregulation of FASN and ACC but not the decrease in ABCG1 or CYP7A1 mRNA levels (fig 5E). These data suggest that huntingtin can regulate the expression of LXR target genes in vivo. LXR agonist rescue of FASN and ACC mRNA expression in huntingtin knockdown zebrafish suggests that huntingtin may regulate these genes through LXR dependent pathways. However, huntingtin may regulate ABCG1 and CYP7A1 expression through LXR independent pathways since treatment with LXR agonist could not rescue their expression in huntingtin knockdown zebrafish.

## DISCUSSION

Huntingtin plays a functional role in regulation of transcription both in its wild-type and mutant form. Microarray studies in cell and mouse models of HD and in human HD brains have revealed widespread dysregulation of transcriptional pathways. In addition, studies of specific transcription factors have catalogued a number of transcription factors able to bind to huntingtin with both dependency and independency on poly-Q length. These include CA150, CREB, COOH terminal binding protein, nuclear receptor co-repressor (N-CoR), p53, RNA polymerase associated protein 30 (RAP30) and Sp1.^5^ ^10^^–^13 32 A key function of wild-type huntingtin in the modulation of REST/NRSF transcription has also been described.^16^

In this study, we have identified several members of the nuclear receptor superfamily that are able to interact with huntingtin. The nuclear receptor superfamily can be divided into six subfamilies based on the evolution of the two well conserved domains of NRs, the DBD and LBD.^33^ Huntingtin interactors—LXRα, PPARγ, VDR and TRα1—are all members of subfamily 1 and function as heterodimers with a common binding partner, RXR. Interestingly, we found RXRα does not bind to huntingtin. We narrowed down the region of LXR that binds to huntingtin to be the LBD. Addition of LXR agonist, however, has no obvious effect on the binding of huntingtin to LXR as assessed by immunoprecipitation (unpublished data). The LBD is well conserved between LXRα, PPARγ, VDR and TRα1, the amino acid identity being 30–38%. The amino acid identity between LXRα and RXRα is less well conserved being <25%. We also found the LBD of PPARγ was able to bind to huntingtin (data not shown). Based on this finding and the sequence homology, it is possible that all four nuclear receptors found to bind to huntingtin interact via their LBDs.

Huntingtin has a multitude of binding partners which cluster into several different functional groups including cytoskeletal organisation and biogenesis, signal transduction, synaptic transmission, proteolysis and regulation of transcription or translation. Huntingtin’s ability to bind to nuclear receptors and other transcription factors to regulate transcription of an even greater number of proteins adds another level of regulation to huntingtin’s functional capabilities. We find that expansion of the polyglutamine tract in mutant huntingtin inhibits its binding to nuclear receptors and its ability to activate LXR transcription. The mutant protein misfolds resulting in a conformational change which could block its interaction with LXR and in turn affect its function. Given huntingtin’s multiplicity of binding partners with diverse functions, disruption of its normal function by expansion of the polyglutamine repeat is likely to have an effect on many cellular processes and it will be important to understand the relative contributions of these different processes to the progression of HD.

The LBD of nuclear receptors is required for ligand binding and receptor dimerisation. LXR/RXR heterodimers bind to LXREs in the promoter region of target genes to regulate transcription. In the absence of agonist, the LXR/RXR heterodimer actively inhibits transcription by recruiting co-repressors. Ligand binding induces dissociation of the co-repressors, leading to moderate stimulation of transcription, and then allows recruitment of co-activators causing maximal stimulation of transcription. We show over-expression of wild-type huntingtin increases, while knockout of huntingtin reduces LXR mediated transcription of an LXRE-luciferase reporter. Furthermore, transcription of the LXR target gene ABCA1 is reduced by huntingtin knockout. Therefore, we suggest wild-type huntingtin acts as a co-factor of LXR mediated transcription. Mutant huntingtin is unable to activate LXR mediated transcription, suggesting that expansion of the polyglutamine tract interferes with normal huntingtin co-factor function.

Recently, huntingtin interacting protein 1 (HIP1) was shown to modulate transcriptional activity of NRs. HIP1 significantly repressed transcription when knocked down using a silencing RNA (siRNA) approach and activated transcription when overexpressed.^34^ Therefore, huntingtin’s co-factor function could be mediated by HIP1. We tested this hypothesis using a silencing RNA approach to knockdown HIP1 in HEK cells transiently expressing LXRE-luciferase reporter, LXR and huntingtin. HIP1 knockdown had no effect on the huntingtin activation of LXR mediated transcription, indicating that HIP1 does not mediate huntingtin’s co-factor function (see supplemental fig 2).

Many transcriptional co-activators contain multiple, short LxxLL motifs that adopt a helical structure to bind to a hydrophobic groove on the surface of the LBD.^29^ Huntingtin 588 contains five of these nuclear receptor interaction motifs; however, mutational analysis demonstrated that they were not necessary for the huntingtin-LXR interaction, indicating huntingtin binds to the LBD via a novel mechanism. LxxLL motif independent binding has been observed previously for constitutive coactivator of PPARγ which has four LxxLL motifs, none of which are important for its binding to PPARγ.^35^ A number of transcriptional regulators contain glutamine-rich activating domains that serve as protein–protein interaction domains.^36^ ^37^ Huntingtin also interacts with several transcription factors such as p53, RAP30, RAP74 and Sp1 via its amino terminal proline rich domain.^10^ ^13^ Deletion of either of these domains, however, had no effect on the binding of huntingtin to LXR (data not shown) suggesting huntingtin binds to LXR via a novel mechanism. Our data demonstrates in vitro binding of LXR to huntingtin, shows deletion of different protein–protein interaction domains of huntingtin (LxxLL motifs, polyglutamine and polyproline domains) has no effect on co-factor function, and shows polyglutamine expansion has an effect on co-factor function, suggesting that huntingtin’s regulation of transcription is a direct rather than an indirect effect of huntingtin.

The physiological role of huntingtin’s co-activation of LXR is unknown. LXRα and β play a central role in the transcriptional regulation of lipid and cholesterol homeostasis.^38^ LXRs act as sensors, modifying expression of genes in pathways that govern transport, catabolism and elimination of cholesterol. Knockout of both LXRα and β led to impaired cholesterol homeostasis and neuronal degeneration in the brain.^26^ Mouse models of HD have impaired cholesterol biosynthesis.^19^ Interestingly, mice overexpressing wild-type huntingtin with 18 glutamines have a reversal of this phenotype with a higher activity of the cholesterol biosynthetic pathway compared to wild-type mice, suggesting that wild-type huntingtin normally plays a key role in cholesterol homeostasis. This phenomenon is entirely compatible with our data where we show a role for wild-type huntingtin in the modulation of LXR activity. Since alterations in LXR activity regulate the transcription of cholesterol excretory genes, wild-type huntingtin could be involved in regulation of cholesterol homeostasis both at the biosynthetic and the catabolic level.

Huntingtin is essential for development in mice. Embryos of huntingtin homozygous knockout mice die by day 8.5.^39^ During development the demand for cholesterol in the brain is particularly high. In adult brain, a large proportion of the cholesterol content derives from its accumulation during the early phases of life. In developing zebrafish, we observed a severe phenotype, with reduction of cartilage in the lower jaw, when we knocked down huntingtin. Furthermore, we observed a decrease in the expression of LXR target genes in huntingtin knocked down fish. The cartilage phenotype and reduced LXR target gene expression can be partially rescued by an LXR agonist, suggesting that huntingtin’s function as a transcriptional co-factor at LXR target genes is important in a physiological context. It would be interesting to conduct studies in conditional huntingtin knock-out mice to elucidate further the in vivo role of huntingtin-LXR interaction and its biological relevance. Huntingtin interactions with other transcription factors, including other nuclear receptors, are also likely to be important during development since treatment with LXR agonist only partially rescued the cartilage phenotype and did not rescue expression of all the LXR target genes affected by huntingtin knockdown. Thus, we believe that the rescue of the FASN and ACC expression in the huntingtin deficient zebrafish by the LXR agonist suggests that huntingtin may control the transcription of these genes mainly via LXR. Conversely, huntingtin may regulate expression of ABCG1 and CYP7A1 via multiple targets and this may explain why they were not rescued by the LXR agonist in the huntingtin knockdown zebrafish. In adults, loss of wild-type huntingtin function in HD and potential disruption of cholesterol homeostasis in the brain may be important for the progression of HD since cholesterol is important for synaptogenesis, neurite outgrowth and neurotransmitter release, processes which are seen to be impaired in HD.^40^
